# The effect of diminished metabolic acidosis on thermoregulatory response during exercise

**DOI:** 10.5114/biolsport.2024.129475

**Published:** 2023-09-20

**Authors:** Tomasz Mikulski, Monika Górecka, Jerzy Smorawiński, Krzysztof Rachwalski, Jakub Kryściak, Krystyna Nazar, Andrzej W. Ziemba

**Affiliations:** 1 Clinical and Research Department of Applied Physiology, Mossakowski Medical Research Institute, Polish Academy of Sciences, Warsaw, Poland; 2 Calisia University, Calisia, Poland; 3 University School of Physical Education, Department of Team Sports Games, Poznan, Poland; 4 University School of Physical Education, Department of Physiology and Biochemistry, Poznan, Poland

**Keywords:** Metabolic acidosis, Sodium bicarbonate, Exercise, Sweating, Temperature, Thermoregulation

## Abstract

It was reported that metabolic acidosis inhibits the activity of warm-sensitive hypothalamic neurons. The present study was designed to test the hypothesis that metabolic alkalosis may improve heat loss during intensive exercise in men. Fifteen male subjects aged 22–24 years were submitted to incremental exercise on two randomized occasions one week apart. During the bicarbonate trial exercise was preceded by ingestion of NaHCO_3_ at a dose 250 mg/kg whilst during the placebo trial lactose was administered. Exercise load was increased every 3 min by 30 W until volitional exhaustion. Ambient temperature was kept at 23–24°C and humidity 50–60%. Tympanic and skin temperatures were recorded and the rate of sweating was assayed by humidity measurement of nitrogen flowing through a capsule attached to the mid posterior chest. Total sweat loss was determined by the changes in body mass. Venous blood samples were taken before exercise and at the end of each workload for determination of acid-base parameters. The subjects attained similar maximal workload in the two tests (260 ± 6 W) with heart rate 185 ± 6 beats/min. Blood concentration of hydrogen ions was lower (p < 0.001) in the bicarbonate than in the placebo trial throughout the whole exercise period. There were no significant differences between these tests in tympanic and mean skin temperatures, sweating rate and total sweat loss. The present data showed that in men attenuation of metabolic acidosis by bicarbonate ingestion did not influence thermoregulation during incremental exercise performed until volitional exhaustion, possibly due to too short duration of exertional uncompensated metabolic acidosis.

## INTRODUCTION

Regulation of body temperature during exercise is achieved by both thermal and nonthermal factors. Increased body temperature triggers cutaneous vasodilation and sweating, but also causes hyperventilation which reduces arterial carbon dioxide partial pressure (PaCO_2_) and cerebral blood flow [1]. Katagiri et al. found that metabolic alkalosis produced by sodium bicarbonate ingestion reduced hyperventilation and attenuated hypocapnia-related cerebral hypoperfusion during exercise [2].

The nonthermal factors include stimulation of the thermoregulatory neurons in the hypothalamus by impulses from motor centres of the cerebral cortex (central command) and stimulation of these neurons by afferent pathways originating in mechanoreceptors and metabolic receptors located in muscles. It is mediated by the superfamily of transient receptor potential (TRP) ion channels, which function as cellular sensors and are activated by several stimuli [3–5]. Among them, the transient receptor potential vanilloid (TRPV) sub-family members, namely TRPV1 and TRPV4, are gated by certain lipophilic molecules, extracellular protons (pH) and stimuli such as heat or osmotic pressure changes [6–8]. Heat and metabolic acidosis, the main physiological indicators modified by muscle contraction during exercise, activate TRPV1 in humans [4, 9]. At lower pH values (< 6.0) TRPV1 is directly activated by protons, whilst at the range of pH 6–9 protons modulate TRPV1 channels by sensitizing them to other stimuli [9, 10].

The firing rate of hypothalamic warm sensitive neurons in rodents is reported to be inhibited by metabolic acidosis [11, 12]. It was suggested that this effect may explain the impairment of the thermoregulatory mechanisms in hypercapnic respiratory acidosis, as well as in thermal stress (i.e. exertional heat stroke), which is usually accompanied by metabolic acidosis [13]. It may be speculated that metabolic acidosis induced by intensive exercise also contributes to the elevation of body temperature. This may partly explain the beneficial effect of training on thermoregulation and the relationship between internal body temperature and exercise loads expressed as percentage of maximal oxygen uptake [14]. It seems that the combination of TRPV channels’ polymodal nature and stimulation sensitivity makes them ideal candidates for stress response proteins that merge signalling pathways and adjust intracellular Ca^2+^ levels as a response to induced stress, such as exercise-induced metabolic acidosis [15].

Therefore, the aim of the present study was to test the hypothesis that diminished metabolic acidosis may favourably affect thermoregulation during exhaustive exercise in men. For this purpose body temperature and sweating rate during exercise with increasing intensity were measured in healthy young men after sodium bicarbonate ingestion intended to diminish metabolic acidosis.

## MATERIALS AND METHODS

### Subjects

Fifteen healthy male students (age: 23.4 ± 0.6 years, body mass: 85.4 ± 2.1 kg, height: 184 ± 1.4 cm, VO_2_peak 51 ± 3 mL/kg/min) participated in the study after giving informed consent. They were physically active but did not take part in any regular sports activity. None of them reported lactose, milk or other dairy product intolerance. The subjects were asked to hydrate properly and not to consume alcohol or perform vigorous exercise in the 24 h before testing and to consume no food or beverages (other than water) 2 h before testing. The study protocol was approved by the Ethical Committee of the Medical University in Warsaw (KB/175/2008).

### Study protocol

A double-blind, placebo-controlled design was employed. The subjects performed an incremental exercise test on two occasions separated by a one-week interval. During the *bicarbonate trial* exercise was preceded by ingestion of NaHCO_3_ at a dose 250 mg/kg of body mass, whilst during the *placebo trial* lactose was used. The dose was chosen in order to avoid the side effects of sodium bicarbonate ingestion, which are dose-dependent, whereas it provided the recommended 5–6 mmol/L increase in bicarbonates’ blood concentration [16, 17]. Both substances were given wrapped in a wafer and ingested during 20–30 min. At that time the subjects in both trials drank 0.9–1.0 L of noncarbonated mineral water. The order of the trials was randomized. The NaHCO_3_ ingestion procedure was similar to that described in previous papers [18].

Exercise started 90 min after bicarbonate or placebo ingestion. Room temperature was kept at 23–24°C, humidity at 50–60%, and the subjects were dressed in shorts and shoes only. Exercise commenced at 30 W and thereafter intensity was increased by 30 W every 3 min until volitional exhaustion. Body mass was measured to the nearest 10 g after voiding before exercise and immediately after its cessation. During exercise heart rate (HR), tympanic temperature (Ttymp) and skin temperature (Tsk) were recorded every 3 min. Tympanic temperature was measured using a thermocouple placed directly on the tympanic membrane (Ellab, Copenhagen, Denmark) and Tsk with an infra-red non-contact thermometer with laser alignment RayTemp3 (ETI, UK). Measurements of Tsk were made on the forehead, arm, trunk and thigh. Local sweating rate was assessed on the basis of relative humidity of nitrogen flowing at the rate of 2.0 L/min through the 20.5 cm^2^ capsule fixed close to the centre of the mid posterior chest, as previously described [19]. During exercise the sweating rate increases significantly in all regions of the body, with the exception of the feet and ankles, and the central part of the posterior chest and lower back produce the highest sweat rates over the whole body during exercise [20]. Before NaHCO_3_ or placebo ingestion and then immediately before exercise and at the end of each exercise load venous blood samples were taken through a venous catheter for determination of concentration of hydrogen ions, base excess, ${\text{HCO}}_{3}{}^{-}$, haemoglobin and haematocrit. The relative humidity of ambient air and nitrogen flowing through the capsule were measured with the Rotronic AG Hygrometer-Control 3 (Switzerland) computerized system with a 1% accuracy. Blood analyses were performed with a Cobas b 121 (Roche, Germany) analyser.

### Calculations

Mean Tsk was calculated according to the following equation:

Mean Tsk=0.6 Tsk trunk+0.1 Tsk arm+0.2 Tsk thigh+0.1 Tsk forehead;

Changes in plasma volume (ΔPV) were estimated from changes in blood haematocrit (Htc) and haemoglobin concentration [Hb] using the following formula [21]:

$$\%\Delta \text{PV}=100\left[\left({\text{Hb}}_{0}/{\text{Hb}}_{\text{t}})(1-{\text{Htc}}_{\text{t}})/(1-{\text{Hct}}_{0}\right)\right]-100\%$$

Where subscripts t and 0 denote measurements at time t and at baseline, respectively. The venous Hct values are multiplied by 0.8736 to obtain values close to those of mean value in the whole vascular system.

The threshold of sweating was calculated using the log transformation method [22]. Total sweat loss during exercise was the difference in body mass measured before and immediately after exercise.

### Statistics

Data are presented as mean ± SE, unless otherwise stated. The effects of treatment on thermoregulatory responses to exercise were tested by two-way ANOVA for repeated measures. Subsequent posthoc pairwise comparisons were performed using the Student t-test. The null hypothesis was rejected when p < 0.05. For calculations Statistica version 6 (StatSoft Inc, Tulsa, OK, USA) was used.

## RESULTS

In both trials duration of exercise ranged from 24 to 30 min and the maximal work loads ranged from 240 to 300 W. Maximal work load achieved by subjects during exercise after placebo and NaHCO_3_ was almost identical (260.6 ± 6 and 264.8 ± 8 W, respectively, p > 0.05), as was maximal heart rate at the end of exercise (186 ± 2 and 184 ± 2 beats/min, p > 0.05).

### Blood acid-base status

**FIG. 1:**
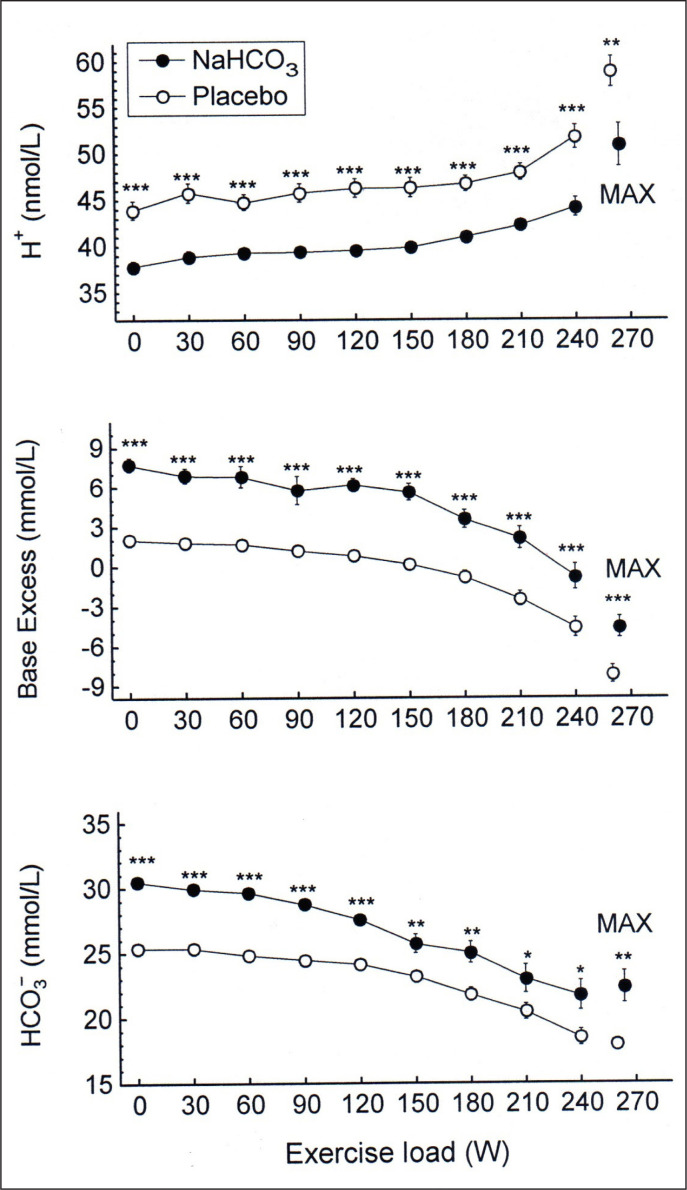
Acid-base balance parameters during exercise in NaHCO_3_ and placebo trials. MAX denotes mean values attained at the maximal exercise load; asterisks indicate a significant effect of NaHCO_3_ ingestion evaluated by two-way ANOVA for repeated measures: * p < 0.05; ** p < 0.01; *** p < 0.001.

As shown in Fig. 1, immediately before exercise blood concentration of H^+^ was lower (by 6.1 ± 1.0 nmol/L, p < 0.001) and blood ${\text{HCO}}_{3}{}^{-}$ and base excess were higher (by 5.1 ± 0.3 mmol/L and 5.1 ± 0.4 mmol/L, respectively, both p < 0.001) in the bicarbonate than in the placebo trial. During exercise the concentration of H^+^ increased in both placebo and bicarbonate trials by 13.0 ± 2.1 nmol/L (p < 0.001) and 12.0 ± 2.2 nmol/L (p < 0.001), respectively, with no difference in the exertional increases between trials (p > 0.05). It corresponded to the decrease in blood pH from 7.34 ± 0.01 to 7.23 ± 0.01 and from 7.41 ± 0.01 to 7.30 ± 0.02 after placebo and bicarbonate ingestion, respectively (both p < 0.001), with no difference in deltas between trials (p > 0.05).

The repeated-measures ANOVA revealed that NaHCO_3_ ingestion had a significant effect on H^+^ concentration in blood (p < 0.001), although there was no significant interaction between effects of treatment and exercise. There was also a significant effect of NaHCO_3_ on blood ${\text{HCO}}_{3}{}^{-}$ (p < 0.001) and base excess (p < 0.001) without significant interactions of the effects of treatment and exercise.

### Thermoregulation

**FIG. 2:**
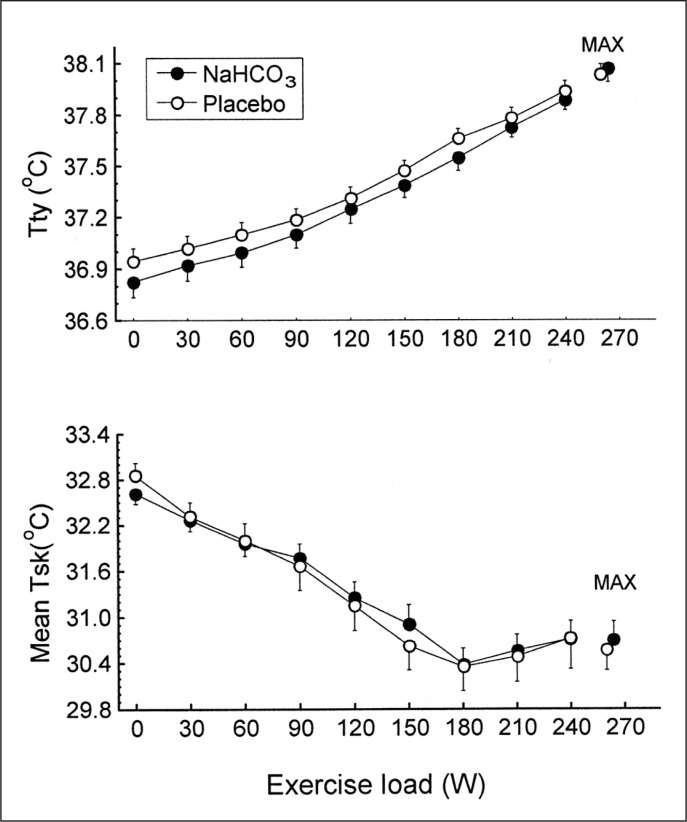
Tympanic (Ttymp) and mean skin (Tsk) temperatures during exercise in NaHCO_3_ and placebo trials. MAX denotes mean values attained at the maximal exercise load.

**FIG. 3:**
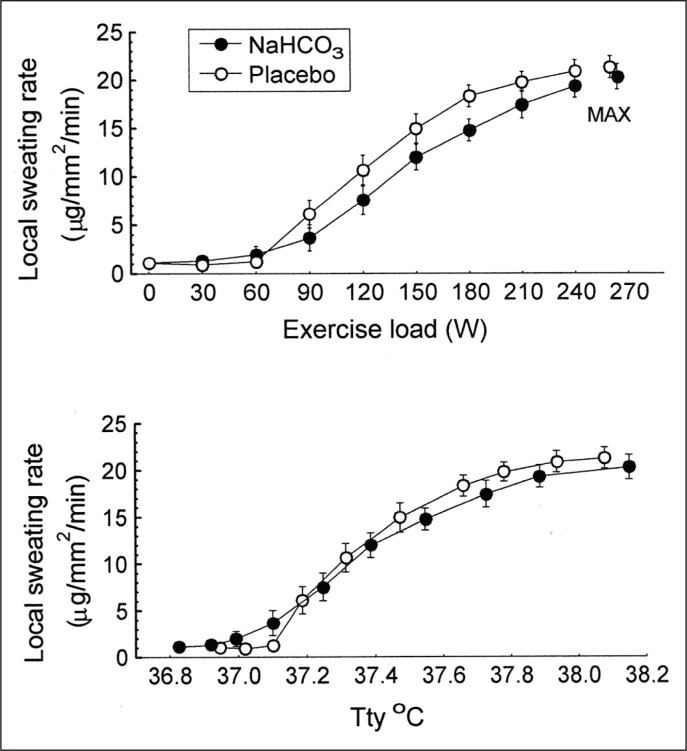
Local sweating rate measured on the mid posterior chestrelated to exercise load and tympanic temperature (Ttymp). MAX denotes mean values attained at the maximal exercise load.

There were no significant differences between trials in the resting tympanic temperature and the rate of its increase during exercise (Fig. 2). The mean exercise-induced increases in Ttymp were 1.1 ± 0.1°C and 1.2 ± 0.1°C in the placebo and bicarbonate trials, respectively, both p < 0.05. The initial values and the time course of mean skin temperature as well as local sweating rate measured on the centre of the mid posterior chest were also similar in both trials (Fig. 3). The threshold of sweating rate increase occurred at exercise loads of 96 ± 10 W and 100 ± 10 W (p > 0.05) and was associated with Ttymp of 37.25 ± 0.06°C and 37.20 ± 0.08°C (p > 0.05) in placebo and NaHCO_3_ trials, respectively. There was no effect of bicarbonate ingestion on the slope of sweating rate increase in relation to Ttymp (p > 0.05). The total sweat loss during exercise calculated from changes in body mass was 0.440 ± 0.080 kg after placebo ingestion and 0.410 ± 0.050 kg (p > 0.05) after bicarbonate treatment.

### Plasma volume

After bicarbonate ingestion, immediately before exercise plasma volume was increased by 2.9 ± 0.9% (p < 0.05), while after placebo it remained practically unchanged (ΔPV = 0.12 ± 1.0%, p > 0.05). During exercise, plasma volume decreased to a similar extent in both trials: by 12.6 ± 1.4% and 11.5 ± 1.6% after bicarbonate and placebo ingestion, respectively (both p < 0.05), with no difference between trials (p > 0.05).

## DISCUSSION

The present study failed to demonstrate any significant effect of diminished metabolic acidosis in blood induced by sodium bicarbonate ingestion on internal body temperature and sweating rate in men during maximal exercise, as well as on sweating threshold in relation to the tympanic temperature. We were not able, therefore, to confirm the hypothesis concerning the role of acid-base balance as a factor contributing to the regulation of body temperature during exercise in humans. However, this finding does not exclude the possibility that metabolic acidosis might adversely influence the heat dissipation mechanism during exercise and/or heat exposure.

Several questions should be considered to interpret the present results. It is not certain whether the hydrogen ion level in blood attained during exercise is high enough to induce the inhibitory effect on hypothalamic warm sensitive neurons or whether metabolic acidosis was maintained for a sufficiently long time to evoke this effect. Comparing metabolic acidosis which occurs during heat stroke with that during maximal exercise without prior alkalization, it appeared that the blood hydrogen ion concentrations were similar [23]. Assuming that metabolic acidosis contributes to the inhibition of heat loss during heat stroke, it seems likely that the exercise-induced metabolic acidosis is sufficient to evoke similar effect [13]. However, it may be speculated that longer duration of metabolic acidosis than that during our exercise test is necessary to influence the thermoregulatory centres. The total duration of exercise, which was 24–30 minutes, was long enough to increase tympanic temperature by more than 1.0°C, but exertional uncompensated metabolic acidosis was present only during the last 6–9 minutes.

Moreover, hypothalamic warm sensitive neurons are partly protected by the blood-brain barrier (BBB), which attenuates the severity of the impact of hydrogen ions. The brain pH results mostly from PaCO_2_ and ${\text{HCO}}_{3}{}^{-}$ concentrations in the brain interstitial fluid. PaCO_2_ is the most potent regulator of cerebral blood flow, whereas alterations in arterial ${\text{HCO}}_{3}{}^{-}$ during acute respiratory acidosis/alkalosis contribute to cerebrovascular acid–base regulation [24, 25]. In both metabolic acidosis and alkalosis in humans changes of pH in cerebrospinal fluid are much smaller than in blood [26].

Lactate transport across the BBB is mediated by the proton-linked monocarboxylate transporter MCT1 that transports one H^+^ for each lactate molecule and saturates near 2.5–3 mmol/L. During exercise or increased nervous activity, lactate production in the brain increases and it should increase efflux of lactate across the BBB. If lactate concentration in blood is also increased, like during exercise, this may not be possible, or even influx can be observed [26]. The extent of brain pH decrease in the present study is hard to determine, but the duration of uncompensated exercise seems to be too short to affect the thermoregulatory neurons.

A similar conclusion was presented by Caldwell et al., who reported unaltered trans-cerebral [${\text{HCO}}_{3}{}^{-}$] exchange during the metabolic acidosis induced by the progressive cycling exercise to exhaustion in humans [27].

However, there are some studies suggesting that BBB integrity might be impaired during exercise, as indicated by the presence of the protein S100β in blood, which is specific for the central nervous system [28, 29]. The factors that may be responsible for transient dysfunction of the BBB during exercise include: development of hyperthermia [30], increase in plasma concentration of adrenaline and acute hypertension [31], increased plasma level of proinflammatory cytokines [32], oxidative stress [33], and changes to the brain serotonin [34]. Interestingly, both TRPV1 and TRPV4 channels are expressed in the subfornical organ area lacking the BBB, which is considered to be the systemic osmosensing region [35, 36].

The transient receptor potential family ion channels, acting as molecular thermometers, are present in many tissues and are influenced by multiple factors, including basic and acidic solutions. Moreover, some other channels, such as the TWIK-related K^+^ channel (TREK1, TREK2) and TWIK-related arachidonic acid stimulated K^+^ channel (TRAAK), are sensitive to both physical and biological stimuli (mechanical forces during pressure changes or cell swelling, lipids, temperature and pH), so the effects of temperature on membrane tension, thickness or curvature may influence channel gating [6, 37]. There is high expression of TREK1, TREK2 and TRAAK in the nervous system, especially in sensory neurons, where they modulate neuronal sensitivity in a highly temperature-dependent manner; channel activity increases with rising temperature [6]. Intracellular and extracellular pH differentially influences the channels: the TREK2 channel is activated by lower pH, whereas TREK1 and TRAAK are inhibited [37].

The time course of these actions and possible interactions during exercise in humans are hard to predict at the current stage of knowledge, constituting an obvious direction for the future research.

Another reason why diminished metabolic acidosis does not modify the thermoregulatory responses to exercise is that the effect of metabolic acidosis occurring during incremental exercise is overwhelmed by several other, nonthermal factors stimulating the hypothalamic heat dissipation centre. Humoral factors, such as an increase of plasma and cerebrospinal osmolality, elevation of sodium ions and a decrease in calcium ion concentrations adversely affect thermoregulation [6, 38–41].

The last question which should be discussed is the possible direct effect of sodium bicarbonate or placebo ingestion prior to exercise on thermoregulation due to the increases in extracellular fluid volume, which may improve thermoregulation [42] and elevation of sodium ion concentration, which in turn may exert an opposite effect [43, 44]. Previous studies demonstrated that hypervolaemia caused by sodium loading using NaHCO_3_ or sodium citrate has a beneficial effect on endurance performance and thermoregulation [45–47]. The beneficial effect of diminished metabolic acidosis on thermoregulation was not taken into consideration in these studies. Our study demonstrated that before exercise plasma volume was increased by approximately 3% after bicarbonate ingestion, indicating that the water pool from which sweat can be drawn was greater in this trial than in the placebo trial, but no differences between the trials in sweating rate or body temperature were found. It might be speculated that either the increase in plasma volume was too small to exert any significant effect on thermoregulation or that the improvement of thermoregulation was prevented by an increase in the availability of sodium ions to the thermoregulatory centres. It should be mentioned, however, that some authors did not find any effect of hypervolaemia on sweating rate or body temperature during exercise [48].

It does not exclude the hypothesis that attenuated metabolic acidosis may improve heat loss during exercise. The observations obtained from cell cultures do not always simply extrapolate to whole organisms, and the total thermoregulatory effect is a complex result of several independent components.

## CONCLUSIONS

The present data showed that in men attenuation of metabolic acidosis by bicarbonate ingestion did not influence thermoregulation during incremental exercise performed until volitional exhaustion, possibly due to too short duration of exertional uncompensated metabolic acidosis.

## Limitations of the study

The experimental design tested a relatively short duration of exercise and mild thermal strain. It does not answer what would happen during prolonged exercise with greater thermal strain or during environmental heat stress.

The focus on male subjects only is obviously a limitation. The majority of physiological phenomena observed in male subjects are simultaneously true for females, whereas possible disturbances caused by the menstrual cycle are avoided.

In the case of application of bicarbonates, possible adverse events should be considered (especially gastrointestinal in chronic supplementation), which limits the applicability of this intervention to help with heat stroke.
